# The regression of myocardial oedema in the first 3 months post ST-elevation MI in patients treated with primary angioplasty

**DOI:** 10.1186/1532-429X-13-S1-P132

**Published:** 2011-02-02

**Authors:** Tom Burchell, Tom Pain, Saidi A Mohiddin, Didier Locca, L Ceri Davies, Steffen E Petersenl, Juerg Schwitter, Anthony Mathur, Mark A Westwood

**Affiliations:** 1 London Chest Hospital, London, UK; 2 Lausanne University Hospital, Lausanne, Switzerland

## Background

Myocardial oedema and salvaged area at risk (ARA) have been described as markers of prognosis and as surrogate markers for clinical trials. The time-course of oedema has been described in a canine infarct model but the in vivo time course following revascularised MI has not been described. CMR is able to characterise oedema in myocardial tissue using T2-weighted sequences. We therefore aimed to determine the time-course of post infarct myocardial oedema using serial CMR imaging.

## Methods

9 patients with acute ST-elevation MI who underwent primary PCI with stent implantation within 12 hours of symptom onset were recruited. Patients were scanned on days 1, 3, 10, 20 and 90 following their PPCI with a 1.5T Philips Achieva (Philips Medical Systems). Images were obtained as continuous short-axis stacks covering the left ventricle with a slice thickness of 8mm and gap of 2mm. Myocardial oedema was assessed at all time points using T2-weighted triple inversion turbo spin echo STIR imaging (TE 80, TR 1667). Scans performed on days 3 and 90 included assessment of scar after administration of a bolus of Gadodoteric acid (0.15 mmol/kg) using a T1-weighted TFE sequence. Image analysis was performed using dedicated software, CMR42 (Circle CVI, Calgary, Canada). Scar and oedema volumes were calculated by manually drawing endocardial and epicardial contours followed by semi-automated selection of normal remote myocardium per slice. The scar and oedema were described as >5SD and >2SD in signal intensity from remote normal myocardium respectively. Values are expressed as a percentage of the LV mass (%LVM).

## Results

**Figure 1:**
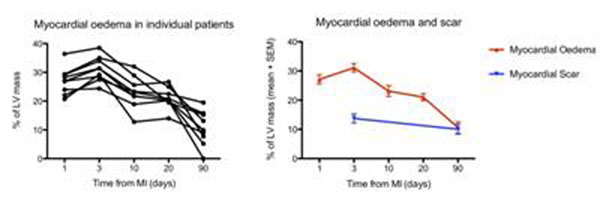


Patient age was 56.6 ± 5.4 years (88% male). Myocardial oedema peaks at day 3 (31.1 ± 4.3%LVM) and is significantly greater than the area of scar (13.7 ± 4.5%LVM) p=<0.0001, with an average salvaged ARA of 17.3 ± 5.7%. There is no difference in oedema between days 1 and 3 or days 10 and 20. There was a reduction of 8% in oedema between days 3 and 10 (p=0.0003, 95% CI 2.8 - 13.1). Oedema persists at 90 days and is the same size as the scar (10.6 ± 6% vs. 10 ± 5.1% p=ns). Figure 1.

## Conclusions

Myocardial oedema post myocardial infarction peaks at day 3 and persists for 90 days. There is no significant change between days 10-20 which may provide the most appropriate time period to assess myocardial oedema. Myocardial oedema may also persist much longer than previously thought.

